# Methods of suicide used by people with cancer: a scoping review protocol

**DOI:** 10.12688/hrbopenres.13886.2

**Published:** 2025-06-06

**Authors:** Doireann Ni Dhalaigh, Zubair Kabir, Fahmi Ismail, Paul Corcoran, Daisy Wiggin, Eugene Cassidy

**Affiliations:** 1 University College Cork, Cork, County Cork, Ireland; 2 Cork University Hospital, Cork, Ireland; 3 National Suicide Research Foundation, Cork, County Cork, Ireland

**Keywords:** Suicide, Cancer

## Abstract

**Background:**

People with cancer are at a higher risk of suicide than the general population. Access to means of suicide is an important volitional risk factor, that if targeted at a population level can reduce incidence of suicide deaths. People with cancer are often prescribed multiple medications that have a high case fatality when taken in overdose and therefore have increased access to specific means of high lethality self-harm. The aim of this review is to examine the methods of suicide used by people with cancer, the study designs used to explore these and what, if any, comparisons have been made to the general population.

**Methods:**

This scoping review will follow JBI scoping review methodology guidelines and be reported according to PRISMA-ScR checklist; a systematic search will be conducted of Embase (Elsevier), CINAHL Plus (EBSCO), PsycINFO (EBSCO), PsychARTICLES(EBSCO), and PubMed (NCBI) databases and grey literature sources. A data collection tool will be specifically designed and piloted independently by two reviewers. Findings will be presented descriptively, graphically, and narratively.

**Results:**

The results of this review will identify the breadth of evidence in relation to methods of suicide used by people with cancer, explore how different methods are defined and categorised, how the topic has been studied, and ascertain if a systematic review is possible.

## Introduction

Suicide risk estimates differ by cancer type, timing, and geographic region of the population studied. Individuals with cancer have a 1.5 to 1.7 times higher risk of suicide than the general population, with elevated risk linked to male sex, older age, recent diagnosis, and certain cancer sites. A matched cohort study found suicide risk was 12.6 times higher in the first week after diagnosis, 3.1 in the first year, and 1.8 thereafter ( Fang & Valdimarsdóttir, 2012).

Access to means of suicide is an important environmental volitional factor ( O’Connor, 2021). Additionally, there is a large evidence base for the reduction of means in the prevention of suicide ( Mann *et al*., 2005; Zalsman *et al*., 2016). For example, legislative interventions to control sales of paracetamol products can significantly reduce incidence of paracetamol overdose ( Corcoran *et al*., 2010; Donohoe *et al*., 2006). Furthermore, evidence indicates that the risk of method substitution, i.e. substituting one suicide method for another, is low ( Cox *et al.*, 2013; Pirkis *et al.*, 2013). The method of suicide chosen can be influenced by many factors, including culture, gender, age, availability and access to means ( Callanan & Davis, 2012; Shah & Buckley, 2011). Across 16 European countries, including Belgium, England, Estonia, Finland, Germany, Hungary, Iceland, Italy (South Tyrol), Luxembourg, the Netherlands, Portugal, Scotland, Slovenia, Spain, and Switzerland, clear gender differences are observed in suicide methods. Hanging is the most frequently used method among both males (54.3%) and females (35.6%). Drug poisoning is the second most common method among females (24.7%) and is used by 8.6% of males ( Värnik *et al*., 2008).

Overdoses using a combination of different medications are associated with higher risk of death ( Daly *et al*., 2020). Pain is a common symptom of cancer and its treatments. The prevalence of pain in cancer is estimated to range from 33% in people who are following curative treatment, 59% in people during cancer treatment and 64% of people with advanced cancer ( Portenoy, 2011). Consequently, many cancer patients will be prescribed a regular regime of pain medication, including opioid medication, particularly in palliative care. People with cancer therefore have a potentially increased access to lethal means of suicide by virtue of increased access to prescribed medications.

In summary, it is evident that the risk of suicide is greater in people with cancer and they have potentially increased access to means of suicide. No review however has examined the evidence available in relation to suicide methods used by this population. This knowledge will be helpful for implementing public health strategies to reduce access to means and to gain more knowledge on lethality of suicidal acts. Scoping review protocols are published to promote transparency, reduce research duplication, and enhance methodological rigour. This protocol serves as a precursor to a scoping review on the same topic.

## Methods

### Reporting

This scoping review protocol has been developed using the best practice guidelines for scoping reviews outlined by ( Peters *et al*., 2022) and is registered on the Open Science Framework. The scoping review will conform to the methodological quality, guidance, and tools as described by ( Pollock *et al*., 2022) and the PRISMA-ScR guidelines ( Tricco *et al*., 2018).

### Eligibility criteria

As scoping reviews aim to map all available literature on a topic, any primary studies quantifying suicide methods among people with cancer will be included ( Munn *et al*., 2018). This may include, but is not limited to, observational studies including cross-sectional, case-control, and cohort studies, either prospective or retrospective. All countries, all ethnicities, all sample sizes, and all publication dates will be eligible for inclusion. Included studies must quantify method(s) of suicide by a population of people with cancer. These criteria were structured using the broad population, concept, context (PCC) framework, as recommended by the Joanna Briggs Institute for scoping reviews, which is a more flexible alternative to the population, intervention, comparator, outcome (PICO) framework typically used in systematic reviews ( Peters *et al*., 2022).

- Population: people with cancer
- Concept: methods of suicide
- Context: N/A

### Review question

Research Question: What is known about the methods of suicide used by people with cancer?

- What study designs have been used?
- How has cancer been defined/diagnosed/operationalised?
- Are different types of cancer examined, such as type, stage, pre or postmortem?
- How have the methods of suicide been defined and categorised?
- How are the methods of suicide classified? i.e. by age, sex, or other variables?
- Have the suicide methods been compared to another population?
- What is the comparator population?
- Is there scope for a systematic review?

### Population

The population will be people with a diagnosis of any malignancy, including single specific cancer types, multiple malignancies, and samples that include a mix of cancer types. This includes any stage, or remission status. This may be diagnosed pre or postmortem and may or not have been known to the decedent. These broad criteria have been chosen to facilitate the inclusion of all research on this population as no scoping reviews have been previously performed.

### Concept

The concept is methods of suicide. This dictates that the participants in question must have died by suicide upon study completion. Differences in nomenclature and definitions of suicide and suicide methods can exist internationally therefore this review will include studies that use any definition of suicide or probable suicide as defined by the authors/country of publication. It is a possible limitation that cultural differences may lead to under-reporting of suicide and suicide methods in some countries, and this is true for all collations of international suicide data. In some countries, where there is insufficient evidence to decide if a death due to self-injury was intentional, for example in the case of an overdose, these are classed as deaths of undetermined intent. As the ultimate outcome is death in both the case of an accidental or intentional act of self-harm, and due to the challenges faced by coroner’s courts in deciphering intent in cases where no suicide note is found, this review will also include deaths of undetermined intent.

Suicide methods will be extracted as reported. This is pertinent as no time limits will be imposed on the studies included. The ICD definitions of methods of suicide have changed significantly since its inception. For example, the ICD 8 includes 7 different categories of suicide methods whereas the ICD 11 includes 83 entries under intentional self-harm under parent category of external causes of morbidity and mortality and this, in addition to differences in data sources such as coroner’s reports or psychological autopsy studies, is likely to influence the range and nomenclature of reported suicide methods ( Stewart *et al.*, 2017; WHO, 2024).

Attempted suicide, parasuicide, or any self-harm where the outcome is not fatal are outside the scope of this review as these conceptually differ from suicide methods that result in death.

### Information sources

Databases will include: Embase (Elsevier), CINAHL Plus (EBSCO), PsycINFO (EBSCO), PsychARTICLES (EBSCO) and PubMed (NCBI). No time or date limits will be applied. Grey search will include Google Scholar, and publications by registries or databases used by included studies. Forward and backward citation searching of included articles will be conducted and author searching via web of science and google scholar will be conducted.

### Search strategy

A sample search strategy is available via the Open Science Framework.

## Study records

### Data management

Results from database searches will be imported to Rayyan to screen papers and remove duplicates. Rayyan will be used to record all decisions made in relation to included and excluded papers and reasons for exclusion. A data collection sheet will be constructed using Microsoft Excel, a copy of this will be made available on Open Science Framework (OSF) upon publication of the scoping review.

### Selection process

Initial screening will be conducted by 2 researchers using Rayyan. During this initial phase titles and abstracts will be screened for relevance to the research question. This screening will be liberal due to the chance that suicide methods may not be mentioned in the title or abstract. To ensure no papers will be missed, any studies that examine suicide and cancer in the title or abstract will be included at this stage. Phase 2 of screening will be conducted by 2 researchers. This will involve full text searching of the main bodies and supplementary material of the papers included during phase 1, to search for information in relation to suicide methods. Phase 3 will include full text screening including supplementary material to determine eligibility according to the inclusion and exclusion criteria. Phase 3 will be carried out independently by 2 researchers, who will be blind to each other’s decisions using Rayyan to communicate results. Any discrepancies will be discussed until consensus is reached or a third reviewer will be consulted to resolve if required. The study selection process, including the numbers identified in the initial search, following abstract, title and full text review, and numbers excluded following full text review will be presented according to PRISMA ScR ( Tricco *et al.*, 2018).

### Data collection process

Studies that meet the eligibility criteria will be marked as included in Rayyan by both reviewers and will then be included in the review. A specific data collection form will be constructed using Microsoft Excel ( Table 1). This tool will be piloted by DND and DW initially to ensure all relevant items are being collected. However, this data collection tool may be subject to change as conducting a scoping review can be an iterative process ( Peters *et al.*, 2022).

**Table 1.** Data collection tool.

title	Author	year	Time span of data collection	Country	Study design	Total participants	Number of participants with cancer & suicide with method described	Definition of suicide reported	Number of comparison population suicides with method described
									
									
Mix of cancer types?	Cancer location	Cancer stage	How was cancer diagnosed?	Suicide method categories	Suicide method definition	How suicide methods diagnosed?	Covariates	Was assisted suicide or euthanasia legal during the time of data collection?	Was suicide risk of cancer population recorded?
									

### Data items

The data items that will be sought are year(s) of data collection, location of study, study design, total number of participants, type(s) of cancer, stage of cancer (i.e. remission, terminal, unknown), number of participants in cancer population and control or comparison population (if applicable), and methods of suicide used by cancer and control/comparison population, definition of suicide methods used, method to ascertain cancer diagnosis, how were suicide methods ascertained, was assisted suicide or euthanasia legal during the time of data collection?, suicide risk of cancer population recorded (y/n), these items may be edited and/or additional items may be added pending piloting of the data collection tool.

### Outcomes and prioritisation

- The main outcome is the categories used for suicide methods.
- The number of studies by study design
- The number of studies that use a comparator population (scope for systematic review)

### Data synthesis

Data will be presented graphically, narratively and in tables as appropriate to the complexity of data items. Descriptive statistics in conjunction with graphical representations may be used to illustrate findings in relation to suicide method categories and study designs. It may be possible to conduct basic descriptive statistics in relation to study designs used also.

### Implications for research and practice

This scoping review will be used to identify if there is scope to conduct a systematic review of methods of suicide used by people with cancer in comparison to a control population. This can be used to infer if access to medications used by people with cancer may increase the risk of death by intentional overdose in this population or not. This scoping review will identify any gaps in the research and research methodologies used and covariates and confounders accounted for in the current literature.

### Deviations from the protocol

Any deviations from this protocol and the underlying reasons for these will be documented and recorded on the OSF and in the final paper.

## Data Availability

No data are associated with this article. Open Science Framework: PubMed search strategy.docx, https://doi.org/10.17605/OSF.IO/6GJPZ ( Dhalaigh, 2024) Data are available under the terms of the Creative Commons Attribution 4.0 International license (CC-BY 4.0).
