# Supplementary material for: Methods of suicide used by people with cancer: a scoping review protocol
Source: HRB Open Res. 2025 Jun 6;7:31. Originally published 2024 May 21. [Version 2] doi: 10.12688/hrbopenres.13886.2 (PMC12255891; doi:10.12688/hrbopenres.13886.2)
Supplement: Peer review history

# Reviewer report: Reviewer response for version 2

**DOI:** 10.21956/hrbopenres.15593.r47612

**Published:** 2025-07-12

**Authors:** Brenna Emery

**Affiliations:** 1 University of Washington, Seattle, USA

Appreciate the changes. This version retains minor grammatical errors but should not exclude it from indexing otherwise.

Is the study design appropriate for the research question?

No

Is the rationale for, and objectives of, the study clearly described?

No

Are sufficient details of the methods provided to allow replication by others?

No

Are the datasets clearly presented in a useable and accessible format?

No

Reviewer Expertise:

Psychiatry, psycho-oncology, consultation-liaison psychiatry, clinical trial medical monitor, traumatic brain injury

I confirm that I have read this submission and believe that I have an appropriate level of expertise to confirm that it is of an acceptable scientific standard.

# Reviewer report: Reviewer response for version 2

**DOI:** 10.21956/hrbopenres.15593.r47613

**Published:** 2025-06-23

**Authors:** Esther de Vries

**Affiliations:** 1 Pontificia Universidad Javeriana, Bogotá, Bogota, Colombia

I have now read the comments and the protocol, and I have no further comments. Approved.

Is the study design appropriate for the research question?

Yes

Is the rationale for, and objectives of, the study clearly described?

Yes

Are sufficient details of the methods provided to allow replication by others?

Partly

Are the datasets clearly presented in a useable and accessible format?

Partly

Reviewer Expertise:

Cancer epidemiology, end of life research.

I confirm that I have read this submission and believe that I have an appropriate level of expertise to confirm that it is of an acceptable scientific standard.

# Reviewer report: Reviewer response for version 1

**DOI:** 10.21956/hrbopenres.15227.r46931

**Published:** 2025-05-19

**Authors:** Brenna Emery

**Affiliations:** 1 University of Washington, Seattle, USA

-There are a number of overt grammatical and tense issues in this paper that need to be addressed. There are too many to list independently, but themes include:

 --Abstract is in future tense. It should be in past tense

 --Run on sentences, lack of use of appropriate commas

 --In general, stylistically, two sentences in a row should not start with the same phrase, ex page 3 "This knowledge will be helpful..." used twice in a row. 

 ---Table 1: Lack of appropriate capitalization

 -Introduction: Why talk about Ireland? This is not a study about Ireland, or if it is it should be specifically mentioned in the paper. There is a sense this was cobbled together from other works clearly aimed at an Irish population. 

 -Methods:

 --You do not need to explain what a scoping review is. Just state your methods.

 --"RQ"- means what?

 --Frequent use of future tense, again

 --I am not used to seeing a "Concept" section in a paper under methods, perhaps Discussion. Perhaps this is cultural.   

 General feedback: What I see here is an interesting idea but not complete paper. If the goal is to write a paper about making a protocol then you need a methods ABOUT generating that protocol, not protocol itself. If you ARE going to do that search then do it and publish the results.  There is mention of verification but nothing about how it's supposed to be done.  I would not personally view this as novel information as it stands. It is a review of suicide risk in patients with cancer and a grant or research proposal.

Is the study design appropriate for the research question?

No

Is the rationale for, and objectives of, the study clearly described?

No

Are sufficient details of the methods provided to allow replication by others?

No

Are the datasets clearly presented in a useable and accessible format?

No

Reviewer Expertise:

Psychiatry, psycho-oncology, consultation-liaison psychiatry, clinical trial medical monitor, traumatic brain injury

I confirm that I have read this submission and believe that I have an appropriate level of expertise to state that I do not consider it to be of an acceptable scientific standard, for reasons outlined above.

# Response

**Published:** 2025-05-29

**Authors:** Doireann Ni Dhalaigh

**Affiliations:** University College Cork, Not Applicable, Cork, Ireland

Thank you for taking the time to review, which has been very helpful. We have addressed each point in detail below and made appropriate revisions, which are tracked in the manuscript. 

**Comment: **There are a number of overt grammatical and tense issues in this paper that need to be addressed. There are too many to list independently, but themes include: --Run on sentences, lack of use of appropriate commas

**Response: **We appreciate this observation and have conducted a full proofread of the manuscript. We have revised the text for clarity, improved sentence structure, and corrected punctuation and tense issues where necessary.

**Comment: **Abstract is in future tense. It should be in past tense

**Response: **As this is a protocol rather than a completed review, the use of future tense in the abstract and methods sections is standard practice and aligns with PRISMA-P and PRISMA-ScR guidance.

**Comment: **Two sentences in a row start with the same phrase (“This knowledge will be helpful…”). Table 1 lacks appropriate capitalization

**Response: **Thank you for highlighting this. We have revised the repetitive phrasing in the introduction for improved readability. Table 1 has been updated to ensure consistent and appropriate capitalisation.

**Comment: **Why talk about Ireland? This is not a study about Ireland… It seems cobbled together from other works aimed at an Irish audience.

**Response: **While the protocol is published on an Irish platform and includes Irish data to illustrate real-world examples, the scope of the review is international. The introduction has been revised to reflect a broader, international perspective.

**Comment: **You do not need to explain what a scoping review is. Just state your methods.

**Response: **This was written as per JBI and PRISMA-ScR guidelines; it is good practice to briefly explain the rationale for using a scoping review approach and to define it for readers who may not be familiar with the methodology. Nevertheless, we have shortened this section for conciseness while retaining key rationale and references.

**Comment: **"RQ" – means what?

**Response: **RQ” refers to “Research Question.” The manuscript has been amended to ensure clarity

 I am not used to seeing a "Concept" section in a paper under Methods, perhaps this is cultural.

 Thank you for this observation. The use of a "Concept" section is part of the Population–Concept–Context (PCC) framework recommended by the JBI Manual for Evidence Synthesis for structuring inclusion criteria in scoping reviews. It is an established methodological standard in this field. A brief explanation and citation to clarify this for readers unfamiliar with the framework (on pg. 5)

**Comment: **This is not a complete paper… it reads more like a grant proposal… If you are going to do the search, then do it and publish the results

**Response: **We respectfully note that this is a protocol, and as such, it is not expected to report results according to PRISMA-P guidelines. Protocols are published to promote transparency, reduce research duplication, and enhance methodological rigour. Scoping review protocols are widely published in peer-reviewed journals (e.g., BMJ Open, HRB Open Research). We have clarified the purpose of the protocol in the introduction.

**Comment: **I would not view this as novel information… it is a review of suicide risk in patients with cancer.

**Response: **We respectfully note that this scoping review does not focus on suicide risk, but on suicide methods used by individuals with cancer, a topic that, to our knowledge, has not been systematically reviewed. This focus provides novel insights into means restriction and suicide prevention strategies.

# Reviewer report: Reviewer response for version 1

**DOI:** 10.21956/hrbopenres.15227.r43051

**Published:** 2024-11-18

**Authors:** Esther de Vries

**Affiliations:** 1 Pontificia Universidad Javeriana, Bogotá, Bogota, Colombia

Applause for the authors to undertake this complex exercise in a complex but important topic.

 In general, I see a very thorough protocol. I wonder how the authors will deal with medically-assisted or assisted suicide? Will these be included or not?

 In table 1 there should be a definition of suicide in the particular country and I would recommend listing if assisted dying (euthanasia or assisted suicide) was allowed in the country from which the study came in the study period.

 Will studies that do not only focus on cancer patients but include them be included? The current search strategy on OSF | pubmed search strategy.docx will result in search 2 and 3 in excluding papers if neoplasms/cancer are nor mentioned in the Mesh terms in Pubmed. There may be studies more generally studying methods of suicide in multiple disease groups and these would be excluded.

 On the OSF page I do not see the search strategies for the other databases, which sometimes have another structure. For the review to be replicable, these terms must be reported either now in the protocol (preferable) or adjustments in the final review being published.

 If one of the intentions is to "identifying the proportions of methods used and by whom can give information about the lethality, intent, and opportunities to intervene to reduce death by suicide. ", then not only suicide deaths but also intents should be included? The protocol says that attempted suicides are not included so then only the "successful" ones will be there and how will lethality be assessed?

 Some recommendations from personal experience: it is important to explicitly include work from all languages and also include search terms for databases in other languages. Studies in these areas are often small and LMIC researchers typically do not have funds to publish internationally. So for Spanish and Portuguese speaking countries databases like LiLacs or ScieLo would be important to include, and probably similar databases exist for francophone and Asian languages databases. The screening of the reference lists may provide even more papers than the search terms themselves as keyword selection is highly influential here. Using AI translation tools on the title and abstract works for initial evaluation of those works published in different languages, and using a wide network of students and staff of other backgrounds leads to possibility of reading most languages. 

 Another suggestion is not to do the extraction in excel but in a software that can easily be blinded and in which it is more difficult to erroneously put information in the wrong cells. We recently used RedCap for a smiliar exercise in which it is sometimes difficult to assign outcomes or exposures to predefined categories and blinding of the colleague´s result is so important. 

 Will data be analysed according to cultures/periods of time of collection or perhaps vision of suicide and/or availability of assisted suicide or euthanasia in a country? It is conceivable that the legal status of assisted dying may be of influence of the frecuency and methods of suicide among cancer patients.

Is the study design appropriate for the research question?

Yes

Is the rationale for, and objectives of, the study clearly described?

Yes

Are sufficient details of the methods provided to allow replication by others?

Partly

Are the datasets clearly presented in a useable and accessible format?

Partly

Reviewer Expertise:

Cancer epidemiology, end of life research.

I confirm that I have read this submission and believe that I have an appropriate level of expertise to confirm that it is of an acceptable scientific standard, however I have significant reservations, as outlined above.

# Response

**Published:** 2025-05-29

**Authors:** Doireann Ni Dhalaigh

**Affiliations:** University College Cork, Not Applicable, Cork, Ireland

Sincere thanks for taking the time to review this protocol and for the constructive feedback. Your comments significantly improved the clarity, rigour, and overall quality of our manuscript. We have addressed each point in detail below and made appropriate revisions, which are tracked in the manuscript.

**Comment: **Applause for the authors to undertake this complex exercise in a complex but important topic.

**Response: **Thank you

**Comment: **In general, I see a very thorough protocol. I wonder how the authors will deal with medically assisted or assisted suicide? Will these be included or not?

**Response: **It is not our intention to include medically assisted suicides due to conceptual differences and lack of relevance to the research question, ie methods of suicide. Agreed that there may be some overlap, ie potentially reduced number of poisoning deaths. However, we feel that it would be a separate research question deserving of its own review/investigation. We will note however during which studies assisted suicide / euthanasia was legalised as per your helpful suggestion.

**Comment: **In table 1 there should be a definition of suicide in the particular country, and I would recommend listing if assisted dying (euthanasia or assisted suicide) was allowed in the country from which the study came in the study period.

**Response: **Added to table 1 on page 7, which will provide important context for interpreting findings.

 1. Definition of suicide reported included in table 1 and 

 2. Assisted suicide or euthanasia legal during the time of data collection?

**Comment: **Will studies that do not only focus on cancer patients but include them be included? The current search strategy on OSF | PubMed search strategy.docx will result in search 2 and 3 in excluding papers if neoplasms/cancer are nor mentioned in the Mesh terms in PubMed. There may be studies more generally studying methods of suicide in multiple disease groups and these would be excluded.

**Response: **Yes, if the methods of suicide used by the group with cancer are reported facilitates extracting quantitative data for this group only. i.e. if the different disease groups are reported together as 1 group, it will not be included. Therefore, this search will be rerun in PubMed using keywords instead of MESH terms for cancer.

**Comment: **On the OSF page I do not see the search strategies for the other databases, which sometimes have another structure. For the review to be replicable, these terms must be reported either now in the protocol (preferable) or adjustments in the final review being published.

**Response: **Only one database search was required as for the protocol, all searches will be uploaded to the Open Science Framework (OSF) with the final scoping reivew.

**Comment:  **If one of the intentions is to "identifying the proportions of methods used and by whom can give information about the lethality, intent, and opportunities to intervene to reduce death by suicide. ", then not only suicide deaths but also intents should be included? The protocol says that attempted suicides are not included so then only the "successful" ones will be there and how will lethality be assessed?

**Response: **This is a very important point, thank you for highlighting it. We removed the word “intent”. Lethality will be assessed by comparing the methods used to published case fatality rates according to suicide method used.

**Comment: **Some recommendations from personal experience: it is important to explicitly include work from all languages and also include search terms for databases in other languages. Studies in these areas are often small and LMIC researchers typically do not have funds to publish internationally. So, for Spanish and Portuguese speaking countries databases like LiLacs or ScieLo would be important to include, and probably similar databases exist for francophone and Asian languages databases. The screening of the reference lists may provide even more papers than the search terms themselves as keyword selection is highly influential here. Using AI translation tools on the title and abstract works for initial evaluation of those works published in different languages, and using a wide network of students and staff of other backgrounds leads to possibility of reading most languages.

**Response: **Thank you for bringing this up. The team are not aware of how many databases are in existence, we do not have the expertise or resources to conduct a systematic search of all relevant database. Moreover, limited resources would prevent professional translation of included papers, which is a requirement to ensure accurate translation. This will be identified as a limitation in the final scoping review. 

**Comment: **Another suggestion is not to do the extraction in excel but in a software that can easily be blinded and in which it is more difficult to erroneously put information in the wrong cells. We recently used Redcap for a similar exercise in which it is sometimes difficult to assign outcomes or exposures to predefined categories and blinding of the colleague´s result is so important.

**Response: **Thank you for the thoughtful suggestion. We have looked into using Redcap however, due to budgetary constraints, we are unable to use it for this review. However, to ensure accuracy and rigour, two reviewers will extract data independently, and discrepancies will be resolved through consensus.

**Comment: ** Will data be analysed according to cultures/periods of time of collection or perhaps vision of suicide and/or availability of assisted suicide or euthanasia in a country? It is conceivable that the legal status of assisted dying may be of influence of the frequency and methods of suicide among cancer patients.

**Response: **We will note during which studies assisted suicide / euthanasia was legalised.
